# CrypTothML: An Integrated Mixed-Solvent Molecular Dynamics Simulation and Machine Learning Approach for Cryptic Site Prediction

**DOI:** 10.3390/ijms26104710

**Published:** 2025-05-14

**Authors:** Chie Motono, Keisuke Yanagisawa, Jun Koseki, Kenichiro Imai

**Affiliations:** 1 Cellular and Molecular Biotechnology Research Institute, National Institute of Advanced Industrial Science and Technology (AIST), Tokyo 135-0064, Japan; jun.koseki@aist.go.jp; 2 Integrated Research Center for Self-Care Technology (IRC-SCT), National Institute of Advanced Industrial Science and Technology (AIST), Tokyo 135-0064, Japan; 3 Department of Computer Science, School of Computing, Institute of Science Tokyo, Tokyo 152-8550, Japan; yanagisawa@comp.isct.ac.jp; 4 Middle Molecule IT-Based Drug Discovery Laboratory (MIDL), Institute of Science Tokyo, Tokyo 152-8550, Japan; 5 Global Research and Development Center for Business by Quantum-AI Technology (G-QuAT), National Institute of Advanced Industrial Science and Technology (AIST), Tsukuba 305-8560, Japan

**Keywords:** cryptic site, mixed-solvent molecular dynamics, machine learning, drug discovery

## Abstract

Cryptic sites, which are transient binding sites that emerge through protein conformational changes upon ligand binding, are valuable targets for drug discovery, particularly for allosteric modulators. However, identifying these sites remains challenging because they are often discovered serendipitously when both ligand-binding (holo) and ligand-free (apo) states are experimentally determined. Here, we introduce CrypTothML, a novel framework that integrates mixed-solvent molecular dynamics (MSMD) simulations and machine learning to predict cryptic sites accurately. CrypTothML first identifies hotspots through MSMD simulations using six chemically diverse probes (benzene, dimethyl-ether, phenol, methyl-imidazole, acetonitrile, and ethylene glycol). A machine learning model then ranks these hotspots based on their likelihood of being cryptic sites, incorporating both hotspot-derived and protein-specific features. Evaluation on a curated dataset demonstrated that CrypTothML outperforms recent machine learning-based methods, achieving an AUC-ROC of 0.88 and successfully identifying cryptic sites missed by other methods. Additionally, CrypTothML ranked cryptic sites as the top prediction more frequently than existing methods. This approach provides a powerful strategy for accelerating drug discovery and designing allosteric drugs.

## 1. Introduction

Proteins exhibit conformational diversity due to their intrinsic dynamics, which can lead to the formation of cryptic sites, which are transient binding pockets that are not detectable in ligand-free (apo) crystal structures [1]. Cryptic sites often play crucial roles in allosteric regulation, where the binding of small molecules at these sites modulates protein function. Given their potential to expand the scope of drug discovery, cryptic sites serve as valuable targets, particularly for allosteric modulators and drugs that target traditionally undruggable proteins [2,3,4].

Many cryptic sites have been discovered serendipitously when both ligand-binding (holo) and ligand-free (apo) states were experimentally determined [2,5]. However, their intentional discovery remains a major challenge. Molecular dynamics (MD) simulations offer a promising approach to detecting dynamically formed cryptic sites [6]. In particular, mixed-solvent MD (MSMD) simulation facilitates the identification of hotspots, which represent ligandable regions on the protein surface and serve as candidate cryptic sites. Hotspots are regions where probe molecules (e.g., benzene and isopropanol) preferentially accumulate during MSMD simulations [7,8,9,10,11,12,13,14,15,16,17,18,19,20]. Several studies have successfully identified cryptic sites using MSMD simulation. Kimura et al. mapped cryptic sites in six out of eight proteins selected from the CryptoSite dataset [21] using 100 ns MD simulations with isopropanol, resorcinol, or acetic acid at 5% concentration, ranking sites based on the free energy calculated from probe occupancy [7]. Schmidt et al. conducted 100–500 ns MSMD simulations with 10% phenol on seven proteins and detected cryptic sites in six cases [8]. Smith and Carlson investigated 12 proteins with diverse conformational changes and identified cryptic sites in seven of them using both conventional and accelerated MSMD simulations with five different probes [11]. These studies demonstrated that MSMD simulations are a useful tool for cryptic site identification. However, MSMD simulation tends to detect multiple hotspots across the protein surface, making it challenging to distinguish hotspots that correspond to cryptic sites from those that do not. Furthermore, the chemical properties of the probe molecules strongly influence hotspot formation, necessitating the use of multiple probes with distinct properties, such as benzene, dimethyl-ether, phenol, methyl-imidazole, acetonitrile, and ethylene glycol. Despite the use of diverse probes, probe occupancy alone is insufficient for reliable cryptic site detection, highlighting the need for additional structural and dynamic information. Indeed, SILCS hotspots, an advanced method combining MSMD simulation with machine learning, has demonstrated improved performance, covering 67 and 89% of the druggable sites within the top 10 and 20 ranked hotspots, respectively [12].

Another challenge is the lack of a standardized definition of cryptic sites. For example, CryptoSite [21] defines cryptic sites as those undetected by FPocket [22] or Concavity [23] when comparing holo and apo structures. However, these criteria do not always account for the full spectrum of cryptic sites, as some apo structures may still accommodate ligands without steric clashes [24].

To address these challenges, we recently developed CrypToth [25], which combines MSMD simulation and topological data analysis to predict cryptic sites. However, CrypToth incurs a considerable computational cost in exchange for its accurate cryptic site prediction. To expand the scope of drug discovery targets, it is necessary to perform cryptic site prediction on a large number of proteins. This requires a machine learning-based prediction method that is both computationally efficient and highly accurate and can also be used as a pre-screening step prior to applying CrypToth. To this end, we constructed a refined dataset with a clearer definition of cryptic sites and developed CrypTothML, a machine learning-based framework that integrates MSMD-derived hotspot features with protein-derived features for cryptic site identification. By ranking hotspots based on their likelihood of being cryptic sites, CrypTothML enables more accurate cryptic site prediction. CrypTothML outperformed existing state-of-the-art machine-learning-based methods for cryptic site prediction.

## 2. Results

### 2.1. Workflow of CrypTothML

In this study, we developed CrypTothML, a cryptic site detection method integrating hotspot identification using MSMD simulations with six different probe molecules and machine learning.

CrypTothML follows the workflow outlined below. First, MSMD simulations with six different probe molecules are conducted to identify multiple hotspots where probe molecules frequently accumulate, representing ligandable regions on the protein surface. Next, hotspot-derived and protein surface-derived features were extracted from each hotspot. Finally, a machine learning model differentiates hotspots corresponding to cryptic sites (referred to as cryptic hotspots) from all detected hotspots by assessing their likelihood of being cryptic sites, represented as probability scores.

**Figure 1:**
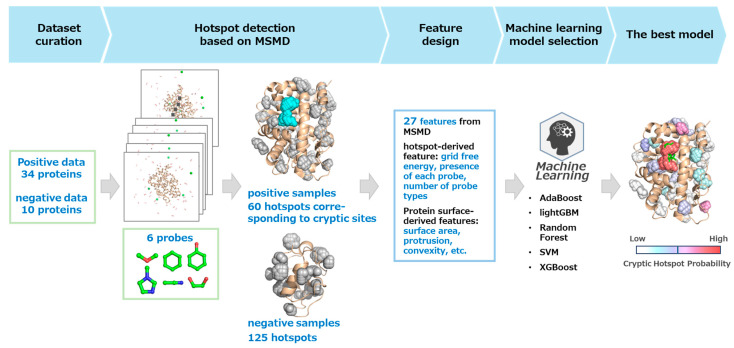
Workflow of CrypTothML development. Overview of the CrypTothML development process, including dataset construction, mixed-solvent molecular dynamics (MSMD) simulations with six probe molecules, machine learning model training.

The workflow for the development of CrypTothML is as follows. We first carefully curated the dataset based on a clear definition of cryptic sites, selecting 34 proteins known to contain cryptic sites and 10 proteins confirmed to lack them from previous studies (Table S1; refer to Materials and Methods 4.1). We then conducted MSMD simulations using six different probe molecules on these proteins. From these simulations, we identified 60 positive hotspots (cryptic hotspots) from 34 proteins and 125 negative hotspots from the 10 proteins, which were used to construct a dataset for machine learning. Next, hotspot-derived and protein surface-derived features were extracted from each hotspot, and multiple machine learning models were trained using various algorithms. Finally, the model with the highest predictive performance was selected as the machine learning component of CrypTothML (Figure 1).

### 2.2. Detection of Hotspots from MSMD Simulations

**Figure 2:**
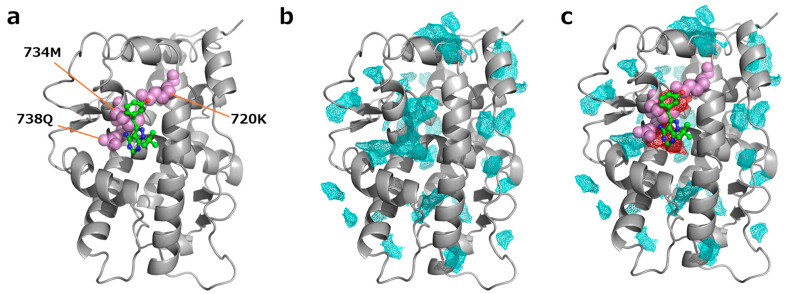
MSMD-derived hotspots in androgen receptor, apo structure (PDB ID: 2am9). (**a**). An example of a cryptic site as defined in this study. The ligand of the holo structure (PDB ID: 2piq) is superimposed onto the apo structure and displayed in a ball-and-stick representation. The residues that sterically clash with the ligand (720K, 734M, and 738Q) are shown as magenta spheres. (**b**). The MSMD-derived hotspots. Hotspots are shown in cyan. (**c**). The identified cryptic hotspot (red) closely overlaps with the location of the clashing residues (i.e., the cryptic site). While hotspots were detected at the cryptic site based on probe occupancy from MSMD, numerous other hotspots were also found on the protein surface. It is therefore important to distinguish true cryptic hotspots from non-cryptic hotspots.

The androgen receptor is one of the representative proteins known to possess a cryptic site. As shown in Figure 2a, the 720K, 734M, and 738Q in the apo structure of the androgen receptor were found to be in steric clash with the ligand when a ligand from the holo structure was superimposed onto the apo structure. The three residues were identified as clashing residues based on our predefined criteria (see Methods Section 4.1) and were thus defined as cryptic sites in this study. Indeed, 720K and 734M are involved in side-chain movement to make the cryptic site accessible [26].

To identify hotspots for each target protein, we performed MSMD simulations using six probe molecules: benzene, dimethyl-ether, phenol, methyl-imidazole, acetonitrile, and ethylene glycol. Hotspots were detected from MSMD trajectories by analyzing and clustering the frequency of probe molecule interactions at specific protein surface regions. As shown in Figure 2b, hotspots were observed in various locations on the surface of the apo structure of the androgen receptor. We then identified cryptic hotspots based on the atomic-distance criteria between hotspots, clashing residues, and ligands (see Methods Section 4.3). Notably, the cryptic hotspot overlaps well with the clashing residues and the ligand (Figure 2c).

Although the structural changes required for the cryptic site opening are not always observed during MSMD simulations, hotspots were still located at the cryptic sites, as shown in Figure 2, suggesting that MSMD-based hotspot detection is a promising method for detecting cryptic sites. However, as numerous hotspots were detected, it was necessary to distinguish true cryptic hotspots from non-cryptic hotspots.

To address this concern, we collected 60 cryptic hotspots from 34 proteins known to contain cryptic sites and 125 non-cryptic hotspots from 10 proteins without cryptic sites. These data were used to develop a machine-learning model capable of distinguishing cryptic hotspot from non-cryptic hotspots.

### 2.3. Feature Selection

As the detection of hotspots is based on MSMD simulations with six probes, we incorporated hotspot-derived features, which include information about the probe molecules constituting the hotspots. Additionally, we adopted protein surface-derived features, such as surface area (size), protrusion [27], convexity [27], compactness [27], hydrophobicity [28], and charge density [29] of the protein surface near the hotspots, most of which are also used in CryptoSite [21]. In the existing method, features related to binding pockets, such as pocket scores obtained from pocket search methods, have proven effective [21]. However, since our MSMD simulations are not designed to induce large changes in pocket scores over long timescales, we incorporated root mean square fluctuation (RMSF) as a protein surface-derived feature. This accounts for structural fluctuations, which may still be significant even if large conformational changes do not occur.

To further clarify the effects of probe molecules, we also conducted MD simulations in a water environment and extracted the same set of probe-derived and protein surface-derived features (Table S2).

### 2.4. Development and Evaluation of the Machine Learning Model

To develop a machine learning model for CrypTothML, we evaluated five supervised learning algorithms (Support Vector Machine (SVM), Random Forest, XGBoost, LightGBM, and AdaBoost) using leave-one-out cross-validation (LOOCV) and then selected the best-performing algorithm. The performance metrics for each algorithm are summarized in Table 1. AdaBoost achieved the highest performance in terms of ROC AUC (0.879) and PR AUC (0.804), while XGBoost achieved the 2nd-best ROC AUC (0.830). LightGBM showed the best precision (0.826) and specificity (0.936), although its recall was relatively low (0.550). Random Forest exhibited the highest recall (0.600), but its precision was lower (0.776). We selected AdaBoost as the final model for CrypTothML based on ROC AUC. Additionally, it achieved the well-balanced precision and recall (the second-best F1-score: 0.692) and the highest PR AUC (0.804), demonstrating its robustness to class imbalance.

In addition, we performed feature importance analysis to score each feature in the AdaBoost model. As shown in Figure S1, the top five features contributing to the prediction were charge density, convexity, size (accessible surface area), hydrophobicity, and compactness. Additionally, electrostatic and hydrophobic properties of the protein surface, surface exposure and shape were found to be important for predicting cryptic sites. Although it is difficult to observe the full conformational transition from the apo to the holo state under the conditions of MSMD simulation used in this study, accurate predictions appear to be achieved by capturing subtle changes in surface shape along with surface physicochemical properties.

### 2.5. Performance Comparison with Existing Methods

We compared the predictive performance of CrypTothML with that of two existing methods, PocketMiner [30] and CryptoSite [21], using the hotspot dataset utilized in this study. The comparison was conducted from two perspectives: overall predictive performance and top-N ranking accuracy of cryptic hotspots. PocketMiner and CryptoSite initially made residue-level predictions. The evaluation metrics and results presented below do not directly reflect their original predictive performance but instead represent their performance based on hotspot-level scores converted from their residue-level outputs (see Methods Section 4.6).

To evaluate the overall predictive performance, we assessed CrypTothML, PocketMiner, and CryptoSite using standard classification metrics, including accuracy, precision, recall, F1-score, specificity, ROC AUC, and PR AUC (Table 2). CrypTothML demonstrated the highest predictive performance in all evaluation metrics. These results indicate that CrypTothML not only provides the highest ROC AUC but also maintains a superior balance between precision and recall, making it a more reliable tool for cryptic site prediction.

Next, to further evaluate the performance of cryptic hotspot ranking for each method, we examined how well the cryptic hotspots were ranked among all hotspots. Assessing the top-N ranking performance is particularly important for drug discovery, where researchers typically focus on a small number of top-ranked predictions. Table 3 summarizes the number of proteins in which cryptic hotspots were ranked within the top 1, 3, or 5 for the 34 proteins containing cryptic sites. CrypTothML ranked cryptic hotspots as the top prediction in 12 proteins (35%), within the top 3 in 20 proteins (59%), and within the top 5 in 23 proteins (68%). In comparison, PocketMiner predicted the top-1 ranked cryptic hotspot in only 6 proteins, while CryptoSite achieved top-1 predictions in 9 proteins, demonstrating that CrypTothML prioritizes cryptic sites more accurately. These results highlight CrypTothML’s ability to more accurately rank cryptic sites compared to existing methods. Since researchers often prioritize top-ranked predictions in drug discovery and structural analysis, ranking performance is a crucial factor for determining the practical utility of a method.

## 3. Discussion

We constructed a refined dataset with a clear definition of cryptic sites and developed CrypTothML, a machine learning-based framework that integrates MSMD-derived hotspot information with protein-derived features for cryptic site identification. CrypTothML outperformed existing methods, achieving the highest ROC AUC (0.879) and PR AUC (0.804), indicating its robustness against class imbalances (Table 2). In addition, CrypTothML predicted cryptic hotspots at the top rank in 35% of proteins containing cryptic sites and approximately 60% within the top five. Its ability to accurately rank cryptic hotspots enhances its applicability in practical settings, making it a valuable tool for prioritizing candidates of previously undiscovered binding sites. Furthermore, combining CrypTothML with CrypToth is expected to enable high-precision cryptic site prediction across a broader range of targets, thereby facilitating drug discovery for undruggable targets.

### 3.1. Case Study Analysis: Fascin and Androgen Receptor

**Figure 3:**
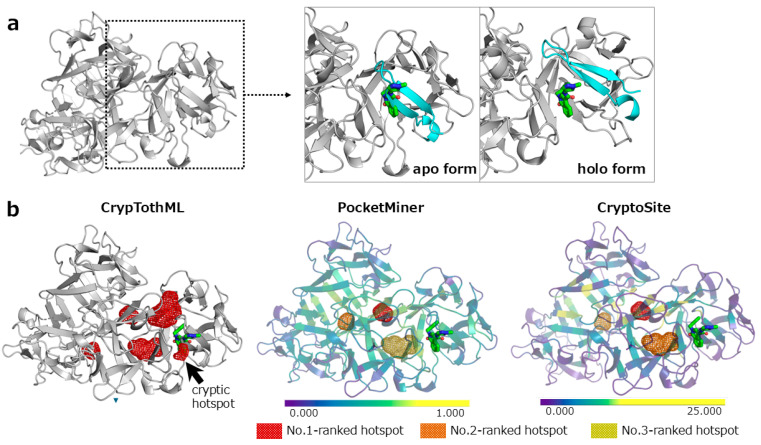
Hotspot ranking for fascin using CrypTothML, PocketMiner, and CryptoSite. (**a**) Molecular structure of apo and holo forms of fascin (apo form: PDB ID: 3p53, holo form: PDB ID: 6i11). The ligand from the holo-structure (a ball-and-stick representation) is superimposed onto the apo structure. The superimposed ligand clashes with a β-strand sterically (colored by cyan), while the β-strand and a loop (colored by cyan) move significantly to form a pocket to accommodate the ligand in the holo structure. (**b**) The top 3 hotspots of each method are color-coded: red for the top-ranked hotspot, orange for the second, and dark yellow for the third. Five hotspots were ranked top 1 with the same probability in CrypTothML. To clarify, the cryptic hotspot is indicated by an arrow. For PocketMiner and CryptoSite, residue-level predictions are visualized as a heatmap on the protein’s cartoon representation. Residues predicted to be cryptic are highlighted in yellow based on the respective threshold scores (0.7 for PocketMiner and 10 for CryptoSite).

To further examine the strengths and limitations of CrypTothML, we illustrate these points using two representative cases: fascin and androgen receptor. Fascin is an actin-binding and bundling protein that undergoes a significant conformational change upon binding to the inhibitor N-(1-methylpyrazol-4-yl)-1-oxidanylidene-2-(phenylmethyl)isoquinoline-4-carboxamide, where a β-sheet and a loop move to form a pocket (Figure 3a) [31]. Owing to this substantial structural rearrangement, fascin presents a particularly challenging case for cryptic site prediction. CrypTothML was the only method that successfully identified a cryptic hotspot in fascin; PocketMiner and CryptoSite failed to identify this site. As CrypTothML performs MSMD simulations within a short timeframe (40 ns), it does not rely on capturing the full pocket-opening process. The detected cryptic hotspot (Figure 3b, red spot in the left panel) partially covered the cryptic site. However, CrypTothML successfully identified the cryptic hotspot. This demonstrates that even without fully reproducing the opening of the cryptic site, short-term MSMD simulations can provide valuable information for cryptic site prediction.

**Figure 4:**
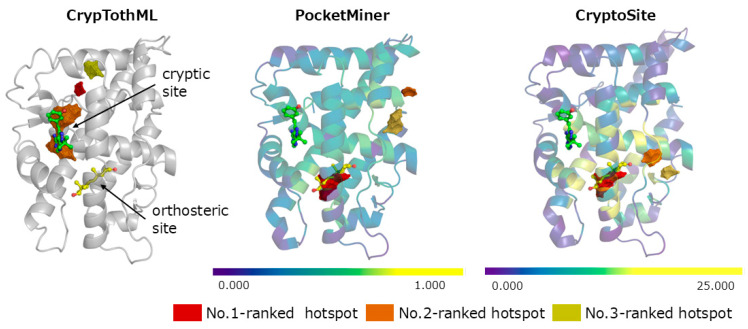
Hotspot ranking of the androgen receptor (PDB ID: 2am9) using CrypTothML, PocketMiner, and CryptoSite. The hotspots ranked by each method are color-coded: red for the top-ranked hotspot, orange for the second, and dark yellow for the third. The ligand from the holo-structure (PDB ID: 2piq) is superimposed onto the apo structure (PDB ID: 2am9). A ligand in the orthosteric binding site is also depicted. For PocketMiner and CryptoSite, residue-level predictions are visualized as a heatmap on the protein’s cartoon representation. Residues predicted to be cryptic are highlighted in yellow based on the respective threshold scores (0.7 for PocketMiner and 10 for CryptoSite).

The androgen receptor (PDB ID: 2am9) contains both cryptic and orthosteric binding sites [26]. As hotspots are typically ranked based on occupancy of probe in conventional MSMD methods, orthosteric sites are usually prioritized over cryptic sites in the androgen receptor [32,33]. Therefore, distinguishing the different types of binding sites is crucial for cryptic site prediction. In this context, the androgen receptor serves as an ideal target. In this case, PocketMiner and CryptoSite ranked the orthosteric binding site as the top one (Figure 4) and placed the cryptic sites at the 9th and 4th ranks, respectively. In contrast, CrypTothML ranked the cryptic site as the 2nd and successfully classified the orthosteric site as non-cryptic, ranking it as 19th. These results indicate that CrypTothML is more practical than the existing methods.

### 3.2. Limitations and Future Perspectives

As demonstrated above, CrypTothML outperformed existing methods in distinguishing cryptic hotspots; however, further validation is needed to assess its generalizability due to the limited dataset size and the diversity of cryptic site formation types. For instance, certain cryptic sites arise at protein–protein interaction (PPI) interfaces, as exemplified by PD-L1. PD-L1 interacts with PD-1 to form a heterodimer, and its inhibitors induce PD-L1 homodimerization, during which a cryptic site has been observed at the interaction surface [34,35]. These cryptic sites, which form at PPI interfaces, are often based on shallow grooves that emerge upon interaction. When examined at the monomeric level, they typically exhibit minimal conformational change. To evaluate the generalizability of CrypTothML, we applied the method to the cryptic site of PD-L1. Although several hotspots, including a cryptic hotspot, were detected, the model failed to identify the cryptic hotspot. This result suggests that, due to the limited diversity of the training dataset, the model has not yet learned to recognize atypical cryptic sites formed through PPIs.

While CrypTothML successfully detected the cryptic site in the challenging case of fascin, it also ranked four other hotspots as the top predictions. These hotspots have not been experimentally confirmed as non-cryptic sites, but they may represent false positives. This highlights the limitation of ranking precision, indicating the need for further refinement to reduce the number of false positives.

In this study, we used five machine learning algorithms to build individual models. To explore the possibility of improving predictive performance, we also constructed two types of consensus prediction models: a stacking model that used the predicted probabilities from these five models as input features with logistic regression as the meta-learner and a simple averaging model that computed the mean predicted probability across the five models. The performance of both consensus models was then evaluated. However, neither approach surpassed the single AdaBoost model (stacking model: ROC AUC: 0.830, PR AUC: 0.757, average model: ROC AUC: 0.827, PR AUC: 0.760). To further improve predictive performance, it may be necessary to incorporate new features, such as the evolutionary conservation of amino acid residues that contribute to prediction, as demonstrated in other studies [21].

Additionally, since LOOCV was used for validation due to the small dataset size, the machine learning model may have overfitted to the dataset. To address this concern, we performed 10-fold cross-validation 1000 times using random splits of the data to evaluate the model’s performance. The ROC AUC (0.835 ± 0.019) moderately decreased, but it still outperformed existing methods. This confirms that robust prediction requires a larger-scale dataset. Recently, a large-scale benchmark dataset, CryptoBench [36], has been reported. However, the definition of cryptic sites used in CryptoBench differs from ours in that it does not take residue-ligand clashes in the apo structure into account. Reconstructing a larger-scale dataset aligned with our definition and incorporating cryptic sites that arise at PPI interfaces could enhance the robustness of CrypTothML and broaden its applicability.

## 4. Materials and Methods

### 4.1. Selection of Protein and Dataset

For this study, we constructed a dataset based on previous studies of cryptic site prediction by selecting 46 proteins with cryptic sites as positive data [7,8,9,11,12]. This selection was refined by identifying the proteins where clear steric clashes with ligands were observed in the apo structure. A cryptic site was defined as a region in the apo structure that sterically clashed with a ligand when the apo structure was superimposed onto the corresponding holo structure. Clashing residues were detected by superimposing the apo structure and the holo structure and evaluating the distances between the heavy atoms of the ligand in the holo structure and those of the protein residues in the apo structure. If the distance was less than 2.5 Å, the residue was considered to be in a steric clash with the ligand. After identifying steric clashes, we applied the hotspot identification method using MSMD simulations (Refer Section 4.3) and selected 34 proteins where hotspots overlapped with cryptic sites as the positive protein dataset. The resulting selection of proteins possessing cryptic sites includes the sites formed by multiple types of conformational changes, such as conformational selection and/or induced fit. However, cryptic sites that require induced fit with large conformational changes upon ligand binding were excluded from our dataset through the selection procedure for clashing residues or cryptic hotspots. Indeed, cryptic sites that arise through such large conformational changes cannot be defined based on residues that clash with the ligand. As a result, the positive dataset is primarily composed of cryptic sites that emerge through conformational selection and/or induced fit with relatively small conformational change.

As the negative protein dataset, we selected ten proteins that did not form cryptic sites, primarily collected from the PocketMiner dataset [30]. Among these, six proteins had multiple resolved holo structures, none of which were observed to bind to ligands, thereby providing extensive structural evidence supporting the absence of cryptic sites. The remaining four proteins were characterized by high stability and rigidity. Given that rigid proteins are highly unlikely to form cryptic sites, they were included as negative data. This selection is consistent with the approach used in the previous study [30], in which rigid proteins were identified based on low flexibility and high structural stability and were further validated by MD simulations showing no formation of cryptic pockets. We adopted the same rationale to construct a reliable negative dataset.

This dataset was used for MSMD simulations and machine learning-based cryptic site prediction.

### 4.2. MSMD Simulations

To conduct MSMD simulations, we used the Protein Preparation Wizard in the Schrödinger Suite 2020-4 [37] to complete the missing loops and side chains for all target proteins. During this process, N- and C-termini were capped with N-methylamide and acetyl groups, respectively. Nonprotein molecules were subsequently removed. The RESP charges for the six probe molecules used in this study (benzene, dimethyl-ether, phenol, methyl-imidazole, acetonitrile, and ethylene glycol) were calculated at the HF/6-31G* level using Gaussian16 [38].

The MSMD simulations were conducted according to the protocol implemented in EXPLORER_MSMD [39]. The MSMD system was constructed using PACKMOL 18.169 [40], in which probe molecules were randomly placed around each protein and equilibrated in a solvent (0.25 M), followed by the addition of water molecules. AMBER ff14SB [41], GAFF2 [42], and TIP3P force fields [43] were applied to proteins, probe molecules, and water molecules, respectively. Additionally, a Lennard-Jones force field term (ε_LJ = 10⁻⁶ kcal/mol, R_min = 20 Å) was introduced between the centers of probe molecules to prevent aggregation. The Lennard-Jones potential is an effective function that enhances the self-repulsion term and prevents non-water molecules in the solvent from clustering [44]. Each MSMD simulation was conducted separately for each probe molecule.

Following energy minimization, NVT MD simulations were conducted with harmonic position restraints on the heavy atoms of the solute (force constant: 10 kcal/mol/Å^2^), gradually heating the system to 300 K over 200 ps. Subsequently, NPT MD simulations were performed at 300 K and 10^5^ Pa for 800 ps, gradually removing position restraints. The P-LINCS algorithm [45] was employed to constrain all bond lengths, including those involving hydrogen atoms, using a 2-fs time step (Δt). In this study, 20 independent MSMD simulations of 40 ns each were executed per system (per probe), and the final 20 ns of each trajectory were analyzed for hotspot detection.

### 4.3. Detection of Hotspots Corresponding to Cryptic Sites

To identify hotspots in each MSMD simulation, we first calculated the occupancy of probe-heavy atoms within a 1 Å × 1 Å × 1 Å voxel grid using PMAP [39]. Because the number of heavy atoms varied across different probe molecules, the occupancy values were normalized by dividing the raw occupancy by the number of heavy atoms in each probe molecule.

Next, voxels with an occupancy exceeding 0.0004 were selected and clustered using the DBSCAN algorithm [46] (DBSCAN parameters: ε_DBSCAN = 3.0 Å, min_samples = 7) to identify clusters. The combination of the occupancy threshold and DBSCAN parameters was empirically optimized to yield approximately 20 to 30 hotspots per protein by adjusting the occupancy threshold (0.001–0.0005), ε_DBSCAN (3.0–4.0 Å), and min_samples (7–10). These clusters represent regions where probe molecules preferentially accumulate on the protein surface during MSMD simulations.

After extracting the clusters for each probe, clusters with more than 20% spatial overlap were merged into single hotspots. This integration of multiple probe clusters ensures that the identified hotspots reflect the physicochemical properties of potential binding ligands.

Finally, hotspots corresponding to cryptic sites were selected based on the following criteria:

(1) At least 80% of the voxels in the hotspot were within 3.5 Å of residue atoms that sterically clash with the ligand molecule.
(2) Additionally, at least one voxel in the hotspot was within 4.5 Å of any ligand atom.
(3) Manual inspection confirms its validity.

Using this hotspot identification method, we obtained 60 hotspots corresponding to cryptic sites (positive examples) from 34 proteins with known cryptic sites and 125 hotspots from 10 proteins without cryptic sites (negative examples). These data were used to construct a training dataset for machine learning. Among the 33 proteins, in 16 proteins, one hotspot corresponded to one cryptic site, while in 17 proteins, multiple (40 in total) hotspots corresponded to one cryptic site. In addition, there was one protein with two cryptic sites: in one cryptic site, one hotspot corresponded to the cryptic site, and in another cryptic site, three hotspots corresponded to it. As a result, a total of 60 cryptic hotspots were obtained.

### 4.4. Feature Extraction for Machine Learning

To train the machine learning model, we extracted features from the probe hotspots and the protein surfaces near these hotspots (Table S2). For each probe-derived feature, we calculated the grid free energy (GFE) and the number of probe molecules occupying the hotspots. These features provide information on the energy favorability and degree of probe accumulation in each region.

For the protein surface-derived features, we computed the feature relating to protein surface (accessible surface area (size), protrusion, convexity, compactness), physicochemical properties (hydrophobicity and charge density), and flexibility. The protein surface near a hotspot was defined as the surface of residues with heavy atoms within 4.5 Å of any heavy atom in the hotspot. Accessible surface area, protrusion, convexity, compactness, hydrophobicity, and charge density were computed as trajectory-averaged values over the MSMD simulations.

Protrusion, convexity, and compactness were computed according to the definitions described by Rossi et al. [27]. Specifically, compactness was defined as the average pairwise distance between the closest surface atoms of residues within a patch, reflecting how tightly the residues are packed. Protrusion was calculated as the fraction of residues within a patch whose atoms have fewer than 120 neighboring atoms in an 8–12 Å shell, indicating how much a residue protrudes from the surface. Convexity was calculated as the ratio of solvent-accessible distances between neighboring residues to the expected centroid distances, the degree of outward curvature of the patch surface.

Flexibility was assessed using the average RMSF values obtained from MSMD simulations for each probe molecule. Six different probe molecules were used in the MSMD simulations for each protein, providing six flexibility values for each protein. The structural and physicochemical surface features were extracted from the MSMD trajectories.

To further assess the influence of the probe molecules, we performed MD simulations in pure water and extracted the same set of features under these conditions.

These features were used as input variables to construct a machine learning model for classifying cryptic and non-cryptic sites.

### 4.5. Evaluation of the Optimal Machine Learning Model

To classify whether a given hotspot corresponded to a cryptic site, we trained five supervised machine learning models: SVM, Random Forest, XGBoost, LightGBM, and AdaBoost, using scikit-learn v1.4.2 [47] implementations. The dataset comprised 60 positive hotspots (cryptic sites) and 125 negative hotspots (non-cryptic sites).

Since the dataset size was small, splitting the data into independent training and test sets made it challenging to reliably evaluate model performance. To address this concern, we employed leave-one-out cross-validation (LOOCV), where each model was trained on n–1 hotspots and tested on the remaining hotspot, iterating over all hotspots in the dataset.

Hyperparameter tuning for each algorithm was conducted using Optuna v 4.2.1 [48], a Bayesian optimization framework, with ROC AUC as the objective function. For the SVM model, a radial basis function (RBF) kernel was used. All input features were standardized to zero mean and unit variance prior to model training. The primary evaluation metric was the area under the receiver operating characteristic curve (ROC AUC). The model with the highest ROC AUC was selected as the best-performing model. If multiple models had similar ROC AUC values, we considered the recall and the balance between recall and precision to determine the optimal model.

In addition to the ROC AUC, we calculated the accuracy, precision, recall, F1-score, specificity, and precision-recall AUC (PR AUC). These metrics were used in comparison with the other methods.

The best model selected for CrypTothML was AdaBoost. The model was trained using the following hyperparameters: max_depth = 4, min_samples_leaf = 2, min_samples_split = 15, learning_rate = 0.0112, and n_ estimator = 190.

### 4.6. Comparison with Existing Methods

To compare CrypTothML with existing state-of-the-art machine-learning-based methods for cryptic site prediction, PocketMiner and CryptoSite, we used the datasets from this study and calculated accuracy, precision, recall, F1-score, specificity, ROC AUC, and PR AUC for each method. In CrypTothML, we used probability output as a ranking score. Whereas for CryptoSite and PocketMiner, which provide residue-level prediction scores, we mapped these scores to each hotspot as follows. For each grid point that constitutes a hotspot, nearby protein atoms within 5.0 Å were identified. A hotspot was considered to be in contact with the protein surface if more than 80% of its grid points were within 5.0 Å of at least one protein atom. For such surface-contacting hotspots, the prediction scores of all protein residues within 5.0 Å were collected and averaged to represent the cryptic potential of the hotspot.

Hotspots were then ranked according to these average scores, and classification was based on the threshold recommended for each method: 10.0 for CryptoSite and 0.7 for PocketMiner.

We also compared the top-N ranking accuracy of cryptic hotspots between CrypTothML and the two other methods. We evaluated the top-N ranking performance based on the number of proteins in which at least one cryptic hotspot was ranked within the top 1, 3, or 5 predictions. Each protein has a single cryptic site; however, in several proteins, multiple cryptic hotspots were identified on the cryptic site, resulting in 60 hotspots across 34 proteins. The percentage of proteins with cryptic hotspots ranked within the top-N is calculated as: (Number of proteins with top-N-ranked cryptic hotspots/34) × 100.

The structural visualization for cryptic hotspot comparison was performed using PyMOL version 2.5.7 (Schrödinger, LLC, New York, NY, USA).

## Figures and Tables

**Table 1:** LOOCV results for different machine learning models (AdaBoost, XGBoost, LightGBM, Random Forest, SVM).

	AdaBoost	XGBoost	LightGBM	Random Forest	SVM
Accuracy	0.827	0.811	0.822	0.822	0.789
Precision	0.818	0.755	0.846	0.776	0.756
Recall	0.600	0.617	0.550	0.633	0.517
F1-score	0.692	0.679	0.667	0.697	0.614
Specificity	0.936	0.904	0.952	0.912	0.920
ROC AUC	0.879	0.830	0.827	0.825	0.806
PR AUC	0.804	0.757	0.760	0.762	0.730

**Table 2:** Performance comparison of CrypTothML and existing methods. Comparison of CrypTothML with existing cryptic site prediction methods (PocketMiner and CryptoSite). For the comparison, web servers of PocketMiner and CryptoSite were used.

Method	CrypTothML (AdaBoost, LOOCV)	PocketMiner	CryptoSite
Accuracy	0.827	0.768	0.719
Precision	0.818	0.743	0.583
Recall	0.600	0.433	0.467
F1-Score	0.692	0.547	0.519
Specificity	0.936	0.928	0.840
ROC AUC	0.879	0.821	0.780
PR AUC	0.804	0.659	0.537

**Table 3:** Top-N ranking performance of CrypTothML and existing methods. Comparison of CrypTothML with existing cryptic site prediction methods (PocketMiner and CryptoSite) based on Top-N ranking performance.

Method	Total Number of Proteins Containing Criptic Sites	Number of Proteins Ranked as Top 1	Number of Proteins Ranked Within Top 3	Number of Proteins Ranked Within Top 5
CrypTothML (AdaBoost, LOOCV)	34	12 (35%)	20 (59%)	23 (68%)
PocketMiner	34	6 (18%)	13 (38%)	14 (41%)
CryptoSite	34	9 (26%)	18 (53%)	21 (62%)

## Data Availability

Initial 3D structures of the proteins were downloaded from the Protein Data Bank (PDB). Schrödinger suite 2020-4 (Schrödinger, LLC, New York, NY, USA) was used for protein preparation. We used AmberTools21 and Gaussian16 Rev B.01 for probe preparation. PACKMOL 18.169 and AmberTools18 were used to prepare the MSMD system. GROMACS 2021.5 was used as the MD engine. The CrypTothML used in this study is available at https://github.com/c-motono/CrypTothML.

## Supplementary Materials

The following supporting information can be downloaded at: https://www.mdpi.com/article/10.3390/ijms26104710/s1. References [49,50,51,52] are cited in the Supplementary Materials.

## Abbreviations

The following abbreviations are used in this manuscript:

GAFF2	General AMBER Force Field 2
GFE	Grid Free Energy
LOOCV	Leave-One-Out Cross-Validation
MD	Molecular Dynamics
MSMD	Mixed-Solvent Molecular Dynamics
PDB	Protein Data Bank
RMSF	Root Mean Square Fluctuation
SVM	Support Vector Machine
