# Supplementary material for: CrypTothML: An Integrated Mixed-Solvent Molecular Dynamics Simulation and Machine Learning Approach for Cryptic Site Prediction
Source: Int J Mol Sci. 2025 May 14;26(10):4710. doi: 10.3390/ijms26104710 (PMC12112718; doi:10.3390/ijms26104710)
Supplement: Supplementary file 1 [file ijms-26-04710-s001.zip › ijms-3591300-supplementary.pdf]

Supplementary materials

CrypTothML: An Integrated Mixed-Solvent Molecular Dynamics Simulation and Machine Learning Approach for Cryptic Site Prediction

Chie Motono [1,2*], Keisuke Yanagisawa[3,4], Jun Koseki [1], Kenichiro Imai[1,2,5*]

[1] Cellular and Molecular Biotechnology Research Institute, National Institute of Advanced Industrial Science and Technology (AIST), Tokyo 135-0064, Japan; c-motono@aist.go.jp; jun.koseki@aist.go.jp; kenichiro.imai@aist.go.jp

[2] Integrated Research Center for Self-Care Technology (irc-sct), National Institute of Advanced Industrial Science and Technology (AIST), Tokyo 135-0064, Japan Department of Computer Science, School of Computing, Institute of Science Tokyo, Tokyo 152-8550, Japan; yanagisawa@comp.isct.ac.jp

[3] Department of Computer Science, School of Computing, Institute of Science Tokyo, Tokyo 152-8550, Japan; yanagisawa@comp.isct.ac.jp

[4] Middle Molecule IT-based Drug Discovery Laboratory (MIDL), Institute of Science Tokyo, Tokyo 152-8550, Japan; yanagisawa@comp.isct.ac.jp

[5] Global Research and Development Center for Business by Quantum-AI Technology (G-QuAT), National Institute of Advanced Industrial Science and Technology (AIST), Tsukuba 305-8560, Japan

* Correspondence: c-motono@aist.go.jp; Tel.; +81-50-3522-9475; kenichiro.imai@aist.go.jp; Tel.; +81- 50-3522-9535; kenichiro.imai@aist.go.jp

Table S1. List of 34 proteins containing cryptic sites (positive data) and 10 proteins not containing cryptic sites (negative data). A summary of the protein dataset utilized in the machine learning model for cryptic site prediction. The citation numbers listed in the reference column correspond to those cited in References section of the main text.

Data Class<br>P: positive, N: negative	Protein Name, gene name	PDB ID (apo)	PDB ID (holo)	References
P	serotonin N-acetyltransferase, AANAT	1b6b	1kuv	[11]
P	guanylate kinase, GUK1	1ex6	1gky	[11]
P	angiopoietin-1 receptor, TEK	1fvr	2oo8	[11]
P	exonuclease I, sbcB	1fxx	3hl8	[7]
P	Beta-lactamase TEM, bla	1jwp	1pzo	[11,49,8,10]
P	NPC intracellular cholesterol transporter 2, NPC2	1nep	2hka	[7,49,50]
P	Mitogen-activated protein kinase 14, MAPK14	1ouy	3hl7	[51,52]
P	Thermonuclease, nuc	1tqo	1tr5	[7]
P	Fatty acid-binding protein, liver, FABP1	1tvq	1tw4	[30]
P	β-secretase, BACE-1	1w50	3ixj	[11]
P	Mitogen-activated protein kinase 14, MAPK14	1wbs	3hvc	[7,8]
P	Aldo-keto reductase family 1 member B1, AKR1B1	1x96	4prr	[8]
P	androgen receptor, ar	2am9	2piq	[11]
P	toluene-4-monooxygenase, tmoD	2bf3	3dhh	[7]
P	Pheromone-binding protein	2fjy	2p70	[30]
P	Dihydrofolate reductase type 1 from Tn4003, dfrA	2w9t	2w9s	[30]
P	TETR-like transcriptional regulator LFRR	2wgb	2v57	[7]
P	Phospholipase A2, major isoenzyme, PLA2G1B	3fvj	2b03	[30]
P	Kinesin-like protein KIF11, KIF11	3hqd	1q0b	[7]
P	Ferulic acid decarboxylase	3nx1	3nx2	[30]
P	Chromatin remodeling regulator CECR2, CECR2	3nxb	5v84	[49]
P	Fascin, FSCN1	3p53	6i11	[30]
P	Anti-Methotrexate CDR1-4 Graft VHH	3qxw	3qxv	[30]
P	Saccharopine dehydrogenase [NAD(+), L-lysine-forming], LYS1	3ugk	3uh1	[30]

P	Suppressor of kinetochore protein 1, SKP1	3v7d	3mks	[7]
P	Serine/threonine-protein kinase BSK8, BSK8	4i92	4i94	[30]
P	Oxysterol-binding protein homolog 3, OSH3	4ic4	4inq	[30]
P	Nopaline-binding periplasmic protein, nocT	4p0i	5ota	[30]
P	ABC-type Fe3+ transport system, periplasmic component, afuA	4r72	4r74	[30]
P	sialyltransferase	4v38	4v3b	[30]
P	Retinol-binding protein 1, RBP1	5h9a	6e5l	[30]
P	UTP--glucose-1-phosphate uridylyltransferase, UGP	5nzm	2oeg	[30]
P	ABC transporter periplasmic-binding protein YtfQ	6hb0	6hbd	[30]
P	Cell division protein FtsZ,	6rvm	5xdt	[30]
N	Endothiapepsin, EAPA	1e5o		[30]
N	Cyclin-dependent kinase 2, CDK2	1oit		[30]
N	Disks large homolog 5, DLG5	1uib		[30]
N	Carbonic anhydrase 2,CA2	1yda		[30]
N	Beta-secretase 1, BACE1	2hiz		[30]
N	Bromodomain-containing protein 4, BRD4	6cd4		[30]
N	Gamma-crystallin B, CRYGB	1amm		[30]
N	Immunoglobulin G-binding protein G, spg	1igd		[30]
N	Crambin, THI2	2fd7		[30]
N	Ubiquitin-ribosomal protein eL40 fusion protein, UBA52	4hjk		[30]

Table S2. List of 27 features used in machine learning. A summary of the 27 features utilized in the machine learning model for cryptic site prediction. These features include MSMD-derived hotspot related properties and protein surface characteristics, capturing both ligand-binding tendencies and structural attributes.

Feature	Description
Hotspot-derived features	
probe_number	Number of probe types constituting the hotspot
probe_benzene_in_hotspot	Presence of benzene in the hotspot (1 = yes, 0 = no)
probe_ dimethyl-ether_in_hotspot	Presence of dimethyl-ether in the hotspot (1 = yes, 0 = no)
probe_phenol_in_hotspot	Presence of phenol in the hotspot (1 = yes, 0 = no)
probe_methyl-imidazole_in_hotspot	Presence of methyl-imidazole in the hotspot (1 = yes, 0 = no)
probe_acetonitrile_in_hotspot	Presence of acetonitrile in the hotspot (1 = yes, 0 = no)
probe_ethylene_glycol_in_hotspot	Presence of ethylene glycol in the hotspot (1 = yes, 0 = no)
gfe	Grid free energy of the hotspots
Protein surface-derived features	
size	Average accessible surface area in the MSMD snaphots
protrusion	Average protrusion in the MSMD snaphots
convexity	Average convexity in the MSMD snaphots
compactness	Average compactness in the MSMD snaphots
hydrophobicity	Average hydrophobicity in the MSMD snaphots
charge density	Average charge_density in the MSMD snaphots
flexibility_benzene	Aaverage RMSF in the snapshots of MSMD with benzene
flexibility_dimethyl-ether	Average RMSF in the snapshots of MSMD with dimethyl-ether
flexibility_phenol	Average RMSF in the snapshots of MSMD with phenol
flexibility_ methyl-imidazole	Average RMSF in the snapshots of MSMD with methyl-imidazole
flexibility_acetonitrile	Average RMSF in the snapshots of MSMD with acetonitrile
flexibility_ethylene glycol	Average RMSF in the snapshots of MSMD with ethylene glycol
size_water	Average accessible surface area in the snaphots of MD in water
protrusion_water	Average protrusion in the snaphots of MD in water
convexity_water	Average convexity in the snaphots of MD in water
compactness_water	Average compactness in the snaphots of MD in water
hydrophobicity_water	Average hydrophobicity in the snaphots of MD in water
charge_density_water	Average charge_density in the snaphots of MD in water
flexibility_water	Average accessible surface area in the snaphots of MD in water

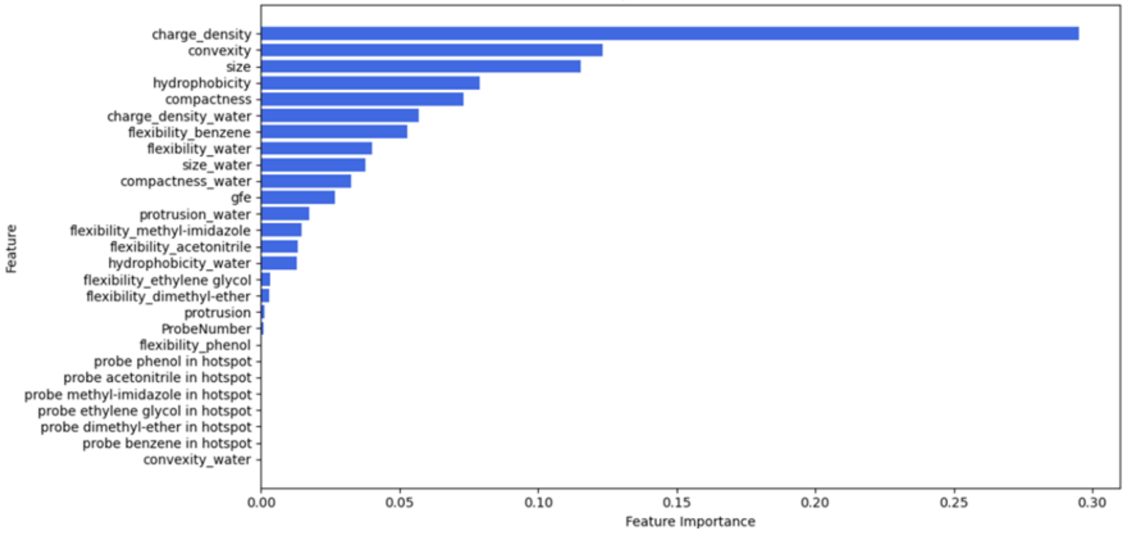

Figure S1. Feature importance in CrypTothML (the AdaBoost model). Feature importance was calculated using the feature_importances_ attribute of the trained AdaBoost model in scikit-learn [47]. This metric is based on the Mean Decrease in Impurity (MDI), which quantifies the total reduction in Gini impurity contributed by each feature across all trees in the ensemble. A higher value indicates greater influence in guiding the decision splits during training. The horizontal bar plot ranks the features by their relative importance in descending order.

References

The citation numbers correspond to those cited in References section of the main text.

7. Kimura, S.R.; Hu, H.P.; Ruvinsky, A.M.; Sherman, W.; Favia, A.D. Deciphering cryptic binding sites on proteins by mixed-solvent Molecular Dynamics. J. Chem. Inf. Model. 2017, 57, 1388–1401. https://doi.org/10.1021/acs.jcim.6b00623.

8. Schmidt, D.; Boehm, M.; McClendon, C.L.; Torella, R.; Gohlke, H. Cosolvent-enhanced sampling and unbiased identification of cryptic pockets suitable for structure-based drug design. J. Chem. Theory Comput. 2019, 15, 3331–3343. https://doi.org/10.1021/acs.jctc.8b01295.

10. Oleinikovas, V.; Saladino, G.; Cossins, B.P.; Gervasio, F.L. Understanding cryptic pocket formation in protein targets by en-hanced sampling simulations. J. Am. Chem. Soc. 2016, 138, 14257–14263. https://doi.org/10.1021/jacs.6b05425.

11. Smith, R.D.; Carlson, H.A. Identification of cryptic binding sites using MixMD with standard and accelerated Molecular Dy-namics. J. Chem. Inf. Model. 2021, 61, 1287–1299. https://doi.org/10.1021/acs.jcim.0c01002.

30. Meller, A.; Ward, M.; Borowsky, J.; Kshirsagar, M.; Lotthammer, J.M.; Oviedo, F.; Ferres, J.L.; Bowman, G.R. Predicting locations of cryptic pockets from single protein structures using the PocketMiner graph neural network. Nat. Commun. 2023, 14, 1177. https://doi.org/10.1038/s41467-023-36699-3.

47. Pedregosa, F.; Varoquaux, G.; Gramfort, A.; Michel, V.; Thirion, B.; Grisel, O.; Blondel, M.; Prettenhofer, P.; Weiss, R.; Dubourg, V.; et al. Scikit-learn: Machine Learning in Python. J. Mach. Learn. Res. 2011, 12, 2825–2830.

49. Zariquiey, F.; Jacoby, E.; Vos, A.; van Vlijmen, H.W.T.; Tresadern, G.; Harvey, J. Divide and Conquer. Pocket-Opening Mixed-Solvent Simulations in the Perspective of Docking Virtual Screening Applications for Drug Discovery. J. Chem. Inf. Model. 2022, 62, 533-543.
50. Bansia, H.; Mahanta, P.; Yennawar, N.H.; Ramakumar, S. Small Glycols Discover Cryptic Pockets on Proteins for Frag-ment-Based Approaches. J. Chem. Inf. Model. 2021, 61, 1322-1333.
51. Tze-Yang, N.; Tan, Y. S. Accelerated Ligand-Mapping Molecular Dynamics Simulations for the Detection of Recalcitrant Cryptic Pockets and Occluded Binding Sites. J. Chem. Theory Comput. 2022, 18, 1969-1981.
52. Tan, Y.S.; Verma, C.S. Straightforward Incorporation of Multiple Ligand Types into Molecular Dynamics Simulations for Efficient Binding Site Detection and Characterization. J. Chem. Theory Comput. 2020, 16, 6633-6644.
